# Mild cognitive impairment has a direct effect on gait speed even when accounting for frailty

**DOI:** 10.1186/s12877-026-07609-1

**Published:** 2026-05-09

**Authors:** Koki Tan, Shigeo Tanabe, Hikaru Kondo, Risako Katada, Miyu Kondo, Kento Katagiri, Sachiko Uehara, Takuma Ii, Taisei Sugiyama, Naoki Mori, Yohei Otaka

**Affiliations:** 1 Department of Rehabilitation Medicine, School of Medicine, Fujita Health University, 1-98 Dengakugakubo, Kutsukake-cho, Toyoake, Aichi Japan; 2 Research Center for Robotic Smart Home & Activity Assistive Technology, Fujita Health University, Toyoake, Aichi Japan; 3 Faculty of Rehabilitation, School of Health Sciences, Fujita Health University, Toyoake, Aichi Japan; 4 Department of Rehabilitation, Fujita Health University Haneda Clinic, Tokyo, Japan

**Keywords:** Older adult, Walking speed, Cognitive dysfunction, Gait analysis, Early detection

## Abstract

**Background:**

Gait—a frequently performed activity of daily living—is thought to reflect multiple dimensions of an individual’s physical and cognitive status. Individuals with frailty or mild cognitive impairment (MCI) show decreased gait speed. However, previous studies have not simultaneously considered both statuses, although they frequently co-occur and may act as confounders. The direct association between frailty and gait is well-understood. In contrast, the association between cognitive decline—independent of physical function—and decreased gait speed, as well as the relationship among these three factors (frailty, cognitive decline, and gait speed), is not fully understood.

**Methods:**

This study examined the effect of MCI on gait speed after accounting for frailty. Older individuals were categorized as (1) frailty with MCI, (2) frailty without MCI, (3) pre-frailty with MCI, (4) pre-frailty without MCI, (5) non-frailty with MCI, and (6) non-frailty without MCI. Frailty was assessed using the Kihon checklist and MCI using the Montreal Cognitive Assessment. Participants completed a 10-m walk test under two conditions: comfortable walking and fast walking. Two types of analyses were conducted: mediation analysis and two-way analysis of covariance (ANCOVA).

**Results:**

Mediation analysis supported independent relationships between frailty and MCI status and gait speed, suggesting a direct association between MCI and gait speed, even when accounting for frailty. In addition, two-way analysis of covariance indicated significant main effects of both frailty and MCI on gait speed, with no significant interaction between them under the two walking conditions.

**Conclusions:**

These findings suggest that the observed association between MCI and gait speed is largely independent from frailty status, providing additional evidence supporting the association between cognitive function and gait performance.

## Background

The global increase in the aging population has become a significant social issue. In 1950, only 5% of the world’s population was aged 65 years and above, which reached 9% in 2020 and is projected to increase to 16% by 2050 [1]. This increase in the aging population is associated with an increase in pre-disability involving the physical, psychological, cognitive, or social aspects [2]. In particular, frailty and mild cognitive impairment (MCI) have garnered increasing attention, with 14% of older individuals aged 65 years and above diagnosed with frailty [2] and 23% with MCI [3]. Clinically, frailty is widely defined as a biological syndrome of decreased reserve and resistance to stressors, often presenting with physical, psychological and social decline [4]. MCI is widely defined as an intermediate status between normal cognition and dementia, characterized by cognitive decline with preserved independence in activities of daily living [5]. These statuses, including pre-frailty as an even earlier stage, represent transitional states between normal aging and physical or cognitive impairment, where early interventions can facilitate reversion to a robust state [6, 7]. Therefore, the early detection of these conditions is increasingly being recognized as a social priority [8].

As one of the approaches to achieving this priority, the measurement of activities frequently performed in daily living has attracted attention. Gait is considered a fundamental component of the activities of daily living and is thought to reflect multiple dimensions of an individual’s physical and cognitive status, including frailty and MCI [9, 10]. For instance, a slow gait speed (< 1 m/s) is included as a screening criterion of frailty [4]. The daily gait speed measured by a smartphone in individuals with frailty was 0.08 m/s less than that in the robust group [11]. Additionally, the gait speed can effectively predict the presence of frailty in older individuals [12]. Regarding MCI, a previous study reported that gait speed was reduced by 0.11 m/s in individuals with MCI compared to that in those with non-MCI [13] because gait is not merely a physical function but rather a complex motor task that requires cognitive domains such as executive function and attention [14]. The decline in gait speed could occur as early as approximately 12 years before the onset of MCI [15].

However, caution should be exercised when interpreting gait speed, particularly in individuals with MCI. Although numerous studies have examined the association between gait speed and MCI, to the best of our knowledge, no previous study has examined gait speed while accounting for frailty status. The direct association between frailty and gait is well understood, as gait speed is a component of frailty screening tools [4]. In contrast, evidence remains unclear whether MCI independently affects gait speed or whether these three factors (frailty, MCI, and gait) are interrelated. In addition, whether frailty and MCI exert a synergistic effect (i.e., an interaction effect) on gait has not yet been investigated. Frailty is a more comprehensive construct encompassing the physical, psychological, and social domains [16]. Therefore, cognitive decline may be associated with non-physical domains of frailty [17], resulting in a partial overlap between the elements of MCI and frailty due to shared underlying mechanisms [18]. Given the increase in co-occurrence of frailty and MCI in older individuals [19], the prevalence of frailty [2], and the significant association between frailty and MCI [20, 21], it is essential to consider both statuses simultaneously when analyzing gait speed.

This study aimed to clarify the associations among gait speed, frailty, and MCI, with a particular emphasis on examining whether adjustment for frailty would eliminate the observed association between MCI and gait speed. A major strength of this study is that we simultaneously analyzed MCI and frailty and their associations with gait. Furthermore, this study examined whether MCI and frailty have synergistic or additive effects on gait. We hypothesized that the association between MCI and gait might be spurious, arising from frailty, which frequently co-occurs with MCI and acts as a confounder.

## Methods

### Study design and participants

This cross-sectional study utilized data collected from older individuals at the Silver Human Resources Centers in Tokyo and Nagoya between June 2024 and November 2024. All participants provided written informed consent before participation, in accordance with the Declaration of Helsinki. The Ethics Review Committee of Fujita Health University approved the study protocol (Approval No. HM23-483). This study was registered in the UMIN Clinical Trial Registry (UMIN000054906).

The inclusion criterion was age ≥ 60 years. The exclusion criteria were as follows: (1) inability to walk independently without a walking aid (such as canes and orthoses), (2) any neurological disease except for MCI, (3) any clinically significant medical or psychiatric condition that interfered with independent daily living, and (4) severe visual and/or hearing impairment.

### Measurement

#### Walking assessment

Participants were instructed to walk on a 10-m walkway under two conditions: comfortable and fast walking. The acceleration and deceleration Sect.  (2 m each) were excluded from the measurement. Using a standard digital stopwatch, a trained examiner measured the time and number of steps for the 6-m walk. The order was the same for all the participants. The participants initially performed a walking trial twice at a self-selected comfortable speed. The participants then performed the trial twice at the fastest speed. Gait speed (m/s) was calculated from the measured data.

#### Frailty assessment

Frailty was assessed using the Kihon checklist (KCL), which was developed by the Japanese Ministry of Health, Labor, and Welfare to identify older individuals at risk of requiring care/support [22]. KCL is a simple self-reporting yes/no survey consisting of 25 questions encompassing multiple domains of physical, social, oral, cognitive, and psychosocial functions. Difficulty with any question is counted as a score in the KCL, with a higher score in each domain indicating a higher risk of requiring support or care. The total score is significantly associated with pre-frailty and frailty and validates the Japanese version of the Cardiovascular Health Study criteria for frailty assessment [23]. Based on this previous study, we classified the participants into three groups according to their total scores: non-frailty (scores 0–3), pre-frailty (scores 4–7), and frailty (scores ≥ 8).

#### Cognitive assessment

Cognitive function was assessed using the Japanese version of Montreal Cognitive Assessment (MoCA-J) (version 8.1) [24, 25]. The assessment yields a total score of 30 points and can be completed in approximately 10 min. According to conventional clinical criteria, participants with a MoCA-J total score < 26 are classified as presence of possible MCI [25]. Note that, as our primary objective was to investigate the association between possible MCI and gait, we did not set other criteria to further differentiate specific MCI subtypes (e.g., amnestic or non-amnestic). All examiners, comprising physical therapists and occupational therapists, have received training on MoCA testing methodologies provided by MoCA Cognition [26].

#### Other assessments

The participants were asked to provide information on their age, sex, current illnesses, medical history, surgical history, and duration of education using a self-administered questionnaire. Body height and weight were measured without shoes.

### Data and statistical analysis

For the preliminary analysis, we categorized participants into two groups: (1) MCI or (2) non-MCI according to MoCA-J score to assess the association between gait speed and MCI status in each walking condition using a t-test. This analysis was conducted to ensure that the results as those obtained in previous studies could be replicated and that a similar population was examined. Demographic characteristics are presented as means and standard deviations (SD). For the walking assessment, representative values for each walking condition (i.e., comfortable and fast) were summarized using the means of two trials.

**Fig. 1:**
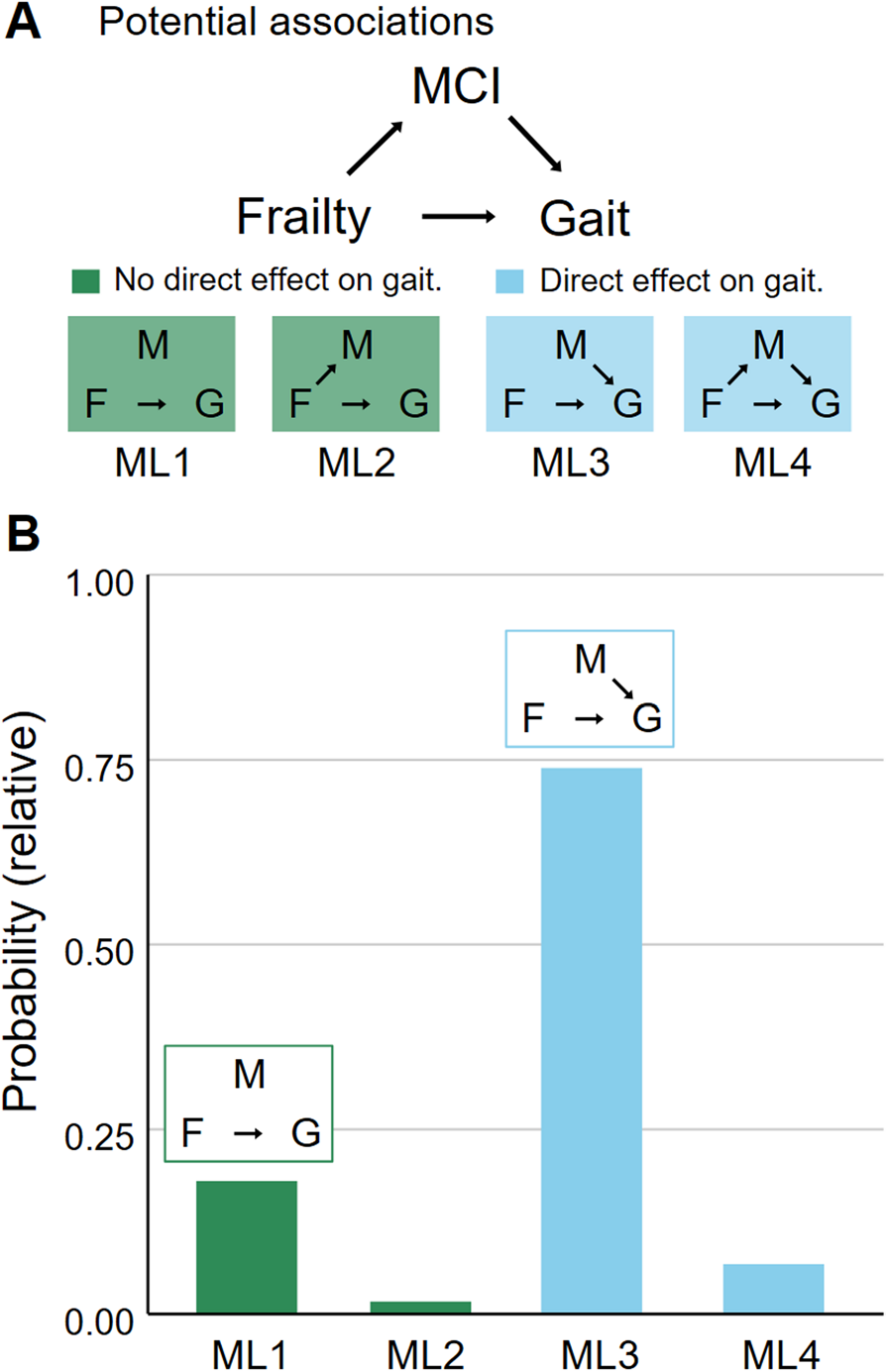
Mediation analysis. Panel **A** indicates the hypothesized mediation model. Four such models were evaluated using Bayesian model selection criteria. Green = mild cognitive impairment (MCI) has no direct effect on gait; Blue = MCI has direct effect. Panel**B** indicates the model that received the strongest support

For the primary analysis, we categorized participants into six groups: (1) frailty with MCI, (2) frailty without MCI, (3) pre-frailty with MCI, (4) pre-frailty without MCI, (5) non-frailty with MCI, and (6) non-frailty without MCI according to KCL and MoCA-J scores. Two types of analyses were then conducted: mediation analysis and two-way analysis of covariance (ANCOVA). For the mediation analysis, we applied the mediation model comparison framework using “bmediatR” package [27] to evaluate the potential associations among frailty, MCI, and gait speed without adjusting for covariates. Given that a direct relationship between frailty and gait has already been established, four models were compared (Fig. 1A), and the model fit and relative support for each association were evaluated using Bayesian model selection criteria. Subsequently, to examine the effect of frailty and MCI status and the interaction effect of these statuses on gait speed, two-way ANCOVA was performed for the two walking conditions. In this analysis, age, sex, and body mass index (BMI) were included as covariates to control for potential confounding factors. When a significant main effect was observed for frailty status (which consisted of three levels), post-hoc pairwise comparisons were performed using estimated marginal means. To strictly control for the family-wise error rate, the Bonferroni correction was applied to post-hoc tests. Effect sizes were evaluated using *Partial η*^*2*^ and *Cohen’s d*, interpreted via established conventions. To align with the one-sided nature of F-tests and prevent interpretative discrepancies, 90% confidence intervals (CIs) were calculated for *Partial η*^*2*^ [28], whereas standard 95% CIs were reported for *Cohen’s d.* Participants with complete data on the primary outcome were included in the analyses. The effects were considered statistically significant at *p* < 0.05. All statistical analyses were performed using R version 4.5.0.

## Results

Initially, 422 older individuals were recruited for this study. One participant was excluded from the analysis because they met the exclusion criterion of being unable to walk without a walking aid. All remaining participants had complete data on the primary outcomes. Consequently, a total of 421 older individuals (213 females) with a mean age of 73.14 (SD 6.11) years participated in this study. The mean comfortable gait speed of the total population was 1.40 m/s (SD 0.23). Among the participants, 215 individuals were classified as possible MCI. Table 1 summarizes the participant characteristics by MCI classification, along with the results of preliminary t-test comparing gait speed between the groups under the two walking conditions (comfortable and fast). The presence of MCI significantly influenced gait speed across all walking conditions (*t*_*(419)*_ = 3.11, *p* = 0.002, *Cohen’s d* = 0.30, 95% CI [0.11, 0.50] for comfortable; *t*_*(419)*_ = 2.69, *p* = 0.007, *Cohen’s d* = 0.26, 95% CI [0.07, 0.45] for fast walking).

**Table 1:** Characteristics by mild cognitive impairment (MCI) status

Outcome	MCI (*n* = 215)	Non-MCI (*n* = 206)	*p*-value
Sex, woman/man	98/117	115/91	
Age, years	74.80 (5.97)	71.38 (5.76)	< 0.001
BMI, kg/m^2^	22.75 (3.19)	22.69 (3.19)	0.852
MoCA-J	22.66 (2.49)	27.41 (1.28)	< 0.001
Kihon checklist	3.33 (3.17)	2.96 (2.66)	0.186
Gait speed, m/s			
Comfortable	1.37 (0.23)	1.43 (0.22)	0.002
Fast	1.82 (0.32)	1.90 (0.28)	0.007

Values are presented as numbers or means (standard deviations). MoCA-J, Japanese version of the Montreal Cognitive Assessment

For the primary analysis, the detailed characteristics of the six groups classified by frailty and MCI status are summarized in Table 2. To address the first objective of the primary analysis to examine whether or not frailty and MCI had independent associations with gait speed, we evaluated the four candidate models using the mediation model comparison framework [27]. Among these models, the ML3 received the strongest support based on the Bayesian model selection criteria (Fig. 1B), which represents the specific theoretical association that statistically best fits the observed data and indicates the most likely relationship among the variables. This model corresponds to the scenario in which frailty and MCI do not influence gait speed through each other. Instead, frailty and MCI exerted direct effects on gait speed primarily via a direct pathway, with negligible mediation by each factor. This suggests that the observed association between MCI and gait speed was independent from the presence of frailty.

**Table 2:** Characteristics by mild cognitive impairment (MCI) and frailty classification

Outcome	Frailty		Pre-frailty		Non-frailty	
	MCI (*n* = 24)	Non-MCI (*n* = 18)	MCI (*n* = 53)	Non-MCI (*n* = 52)	MCI (*n* = 138)	Non-MCI (*n* = 136)
Sex, woman/man	16/8	5/13	25/28	21/31	76/62	65/71
Age, years	76.83 (4.90)	73.28 (5.13)	74.89 (5.46)	71.46 (6.44)	74.42 (6.28)	71.10 (5.56)
BMI, kg/m^2^	21.80 (3.17)	21.65 (4.03)	23.45 (3.04)	21.88 (2.78)	22.64 (3.21)	23.13 (3.15)
MoCA-J	21.83 (2.62)	27.50 (1.47)	22.96 (2.39)	27.38 (1.24)	22.69 (2.48)	27.40 (1.28)
Kihon checklist	10.42 (2.22)	9.22 (1.59)	5.00 (1.07)	4.79 (0.94)	1.46 (1.00)	1.43 (1.09)
Gait speed, m/s						
Comfortable	1.23 (0.29)	1.27 (0.20)	1.32 (0.22)	1.39 (0.21)	1.41 (0.21)	1.47 (0.22)
Fast	1.64 (0.31)	1.68 (0.25)	1.76 (0.34)	1.88 (0.26)	1.88 (0.29)	1.94 (0.27)

Values are presented as numbers or means (standard deviations). MoCA-J, Japanese version of the Montreal Cognitive Assessment

**Fig. 2:**
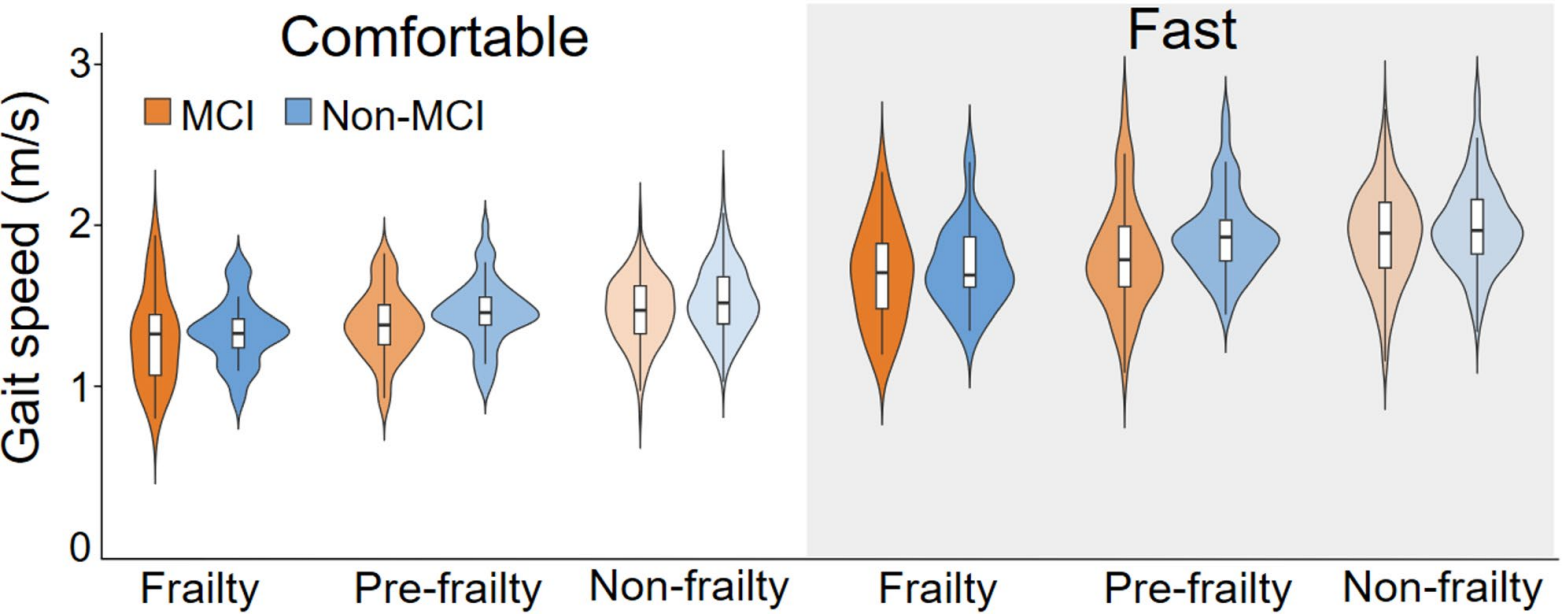
Violin plot of walking speed in individuals with and without frailty and mild cognitive impairment (MCI). Left panel indicates comfortable walking. Right panel indicates fast walking. Orange and blue indicate individuals with MCI and non-MCI, respectively

Subsequently, to examine whether frailty and MCI status have synergistic effects (i.e., an interaction) on gait speed, ANCOVA was performed for the two walking conditions (Fig. 2; Table 3). Even when both factors were evaluated simultaneously, MCI had a significant effect on both comfortable walking (*F*_*(1, 412)*_ = 10.7, *p* = 0.001, *Partial η*^*2*^ = 0.03, 90% CI [0.01, 0.06]) and fast walking (*F*_*(1, 412)*_ = 8.6, *p* = 0.003, *Partial η*^*2*^ = 0.02, 90% CI [0.00, 0.05]). Similarly, frailty had a significant effect on both comfortable (*F*_*(2, 412)*_ = 16.0, *p* < 0.001, *Partial η*^*2*^ = 0.07, 90% CI [0.04, 0.11]) and fast (*F*_*(2, 412)*_ = 15.8, *p* < 0.001, *Partial η*^*2*^ = 0.07, 90% CI [0.03, 0.11]) walking. There were no interaction between MCI and frailty on gait speed in either walking condition (*F*_*(2, 412)*_ = 0.12, *p* = 0.888, *Partial η*^*2*^ < 0.01, 90% CI [0.00, 0.00]) for comfortable; *F*_*(2, 412)*_ = 0.32, *p* = 0.727, *Partial η*^*2*^ < 0.01, 90% CI [0.00, 0.01] for fast walking). Bonferroni-corrected post-hoc tests for comfortable gait speed revealed a stepwise decrease across groups: frailty vs. non-frailty (*t*_*(412)*_ = 5.02, *p* < 0.001, *Cohen’s d* = 0.85, 95% CI [0.51, 1.19]), frailty vs. pre-frailty (*t*_*(412)*_ = 2.55, *p* = 0.033, *Cohen’s d* = 0.47, 95% CI [0.11, 0.84]), and pre-frailty vs. non-frailty (*t(*_*412)*_ = 3.28, *p* = 0.003, *Cohen’s d* = 0.38, 95% CI [0.15, 0.61]). For fast gait speed, the frailty group remained significantly slower than the non-frailty (*t*_*(412)*_ = 4.72, *p* < 0.001, *Cohen’s d* = 0.80, 95% CI [0.46, 1.14]) and pre-frailty (*t*_*(412)*_ = 2.84, *p* = 0.014, *Cohen’s d* = 0.53, 95% CI [0.16, 0.89]) groups, whereas the pre-frailty vs. non-frailty difference was only marginally significant (*t*_*(412)*_ = 2.36, *p* = 0.056, *Cohen’s d* = 0.27, 95% CI [0.04, 0.50]).

**Table 3:** Two-way ANCOVA (Frailty × MCI) in each walking conditions

Factors	Df	F-value	*p*-value	Partial η^2^ [90% CI]
Comfortable				
Frailty	2	16.00	< 0.001	0.07 [0.04, 0.11]
MCI	1	10.70	0.001	0.03 [0.01, 0.06]
Frailty × MCI	2	0.12	0.888	0.00 [0.00, 0.00]
Covariate				
Age	1	10.95	0.001	0.03 [0.01, 0.06]
Sex	1	0.35	0.552	0.00 [0.00, 0.01]
BMI	1	7.17	0.008	0.02 [0.00, 0.04]
Fast				
Frailty	2	15.81	< 0.001	0.07 [0.03, 0.11]
MCI	1	8.58	0.004	0.02 [0.00, 0.05]
Frailty × MCI	2	0.32	0.727	0.00 [0.00, 0.01]
Covariate				
Age	1	28.83	< 0.001	0.07 [0.03, 0.11]
Sex	1	19.23	< 0.001	0.04 [0.02, 0.08]
BMI	1	3.18	0.075	0.00 [0.00, 0.01]

Analysis was adjusted for age, sex, and body mass index. Residual degrees of freedom = 412. A 90% confidence interval was reported for *Partial η*^*2*^, in accordance with methodological recommendations suggesting that a 90% CI is more appropriate given the one-sided nature of F-tests

*ANCOVA* Analysis of covariance, *Df* degrees of freedom, *MCI* Mild cognitive impairment

## Discussion

This study investigated whether MCI influences gait speed after accounting for the presence of frailty. The findings of a simple comparison between MCI and non-MCI groups showed a significant difference in gait speed, and this difference was significant even when frailty and MCI were evaluated simultaneously under two walking conditions (comfortable and fast).

Gait speed differed significantly between individuals with and without MCI under two walking conditions, consistent with previous studies. For instance, usual gait speed is reportedly reduced by early symptoms of cognitive impairment [29]. Moreover, a significant difference in gait speed was observed between older individuals with MCI as defined by MoCA-J and those without MCI [30]. As the present study can be considered to target a population similar to those of previous studies, the multivariate analysis performed herein had a high validity.

To the best of our knowledge, this is the first study to simultaneously examine the associations of MCI and frailty with gait speed. Initially, we hypothesized that the association between MCI and gait might be spurious, arising from frailty, which frequently co-occurs with MCI and acts as a confounder. Regarding frailty, a direct effect on gait speed was observed even when accounting for the influence of MCI using mediation analysis. This finding is consistent with that of previous studies [31, 32] that did not account for the influence of MCI status but demonstrated that frailty is associated with gait characteristics. In addition, our results indicate that MCI had a significant main effect on gait speed directly, even after accounting for the presence of frailty and MCI simultaneously. This finding could be explained by the direct association between gait performance and cognitive function, even in simple single-task walking situations. Previous studies have reported that not only muscle strength weakness [33, 34] but also executive dysfunction is associated with a decline in gait speed among older individuals [14, 35]. Given that MCI is characterized by cognitive impairment, including executive dysfunction [36, 37], our findings provide additional robust evidence to support the association between cognitive function and gait performance.

The absence of an interaction effect is consistent with the model selected in the mediation analysis and can thus be regarded as reasonable. One possible explanation behind this result is the difference in the mechanism underlying the decline in gait speed. In other words, the effects of MCI and frailty are largely independent and additive, rather than synergistic. Although frailty is considered a comprehensive and multidimensional construct, it predominantly encompasses components of physical function [38, 39]. Considering that MCI is a component of cognitive function, frailty and MCI largely represent distinct functional domains. This finding is also consistent with associations, such as the ML3, in which MCI and frailty may share upstream determinants, such as age [19], but do not modify each other’s effects on gait performance. Because MCI and frailty appear to exert independent associations, it is plausible that different gait characteristics may emerge when gait speed is decomposed into more specific gait features. Nonetheless, caution is warranted when interpreting these findings as walking in real-world situations—where higher levels of executive function are typically required [40]—may yield different results. Indeed, a previous meta-analysis indicated that dual-task walking conditions were more effective than single-task walking in detecting MCI [41]. Thus, for clinical implications, the same demonstration should be conducted under various walking situations, including authentic daily walking.

Furthermore, our results provide important quantitative insights regarding the relative effect of frailty versus MCI status on gait speed. Considering the effect size (*Partial η²*) on gait speed, frailty had an approximately twice the value compared to MCI (0.07 vs. 0.03) in single-task walking. As mentioned above paragraph, MCI and frailty have distinct mechanisms that contribute to a decline in gait speed. Although gait is considered a multifaceted function involving various components [42], the current evidence does not clarify which factors contribute more strongly to a decline in gait speed. By demonstrating that the effect size of frailty is larger than that of MCI, our results suggest that frailty is more strongly related to gait speed more than MCI status and contributes to new findings that reveal the extent to which each factor influences gait speed.

The present study has several limitations. First, we used only screening tools (i.e., MoCA-J and KCL) to study general characteristics in a relatively large number of participants. However, a trade-off is lack of diagnostic certainty and discriminability, limiting the generalizability of our findings to specific sub-populations (e.g., amnestic MCI) and cognitive domains (e.g., executive function) [40]. Thus, our findings should be complemented with domain-specific investigations with clinical diagnoses by physicians to verify whether MCI and frailty still remain independent or they actually interact in specific populations. Second, the study was not designed to infer a potential causal structure among frailty, MCI, and gait speed. Therefore, there may be a discrepancy between the statistically preferred model and the actual structure. For instance, in the actual structure, a mediator could be frailty instead of MCI, or the direction of the pathway could be opposite. These alternative possibilities should be examined in a separate study designed for causal inference, such as a longitudinal design that examines temporal precedence among variables [43]. Third, all the study participants lived in urban areas and were still working. The mean gait speed of all our participants (1.40 m/s) was relatively fast compared to that of community-dwelling older Japanese individuals reported in previous large-scale studies (1.25 to 1.29 m/s) [44, 45]. Furthermore, even participants who were classified as co-occurring MCI and frailty in this study exhibited a mean gait speed of 1.23 m/s, which exceeds one of the frailty screening criteria (1.0 m/s) [4]. Given that the absolute gait speed may be influenced by regional and lifestyle factors [46–48], caution should be exercised on the generalizability of our findings. Finally, our walking experiments were conducted exclusively in an experimental setting and did not reflect walking in daily life. While measurements obtained under controlled conditions allow for the collection of high-quality data, it should be noted that such conditions differ from those encountered in real-world daily walking, which often demands more executive functioning and attentional resources [40]. Consequently, our exclusive focus on single-task walking may have underestimated the true association between cognitive decline and everyday gait performance.

In conclusion, the present study demonstrated that MCI has a significant effect on gait speed, even after accounting for the presence of frailty. Our findings suggest that the observed association between MCI and gait speed is independent from frailty, highlighting that cognitive function is directly related to gait characteristics even in natural walking situations, such as a comfortable self-selected walking speed.

## Data Availability

Data will be made available on reasonable request.
